# Plasma eicosapentaenoic acid, a biomarker of fish consumption, is associated with perfluoroalkyl carboxylic acid exposure in residents of Kyoto, Japan: a cross-sectional study

**DOI:** 10.1265/ehpm.22-00302

**Published:** 2023-06-20

**Authors:** Sani Rachman Soleman, Meng Li, Tomoko Fujitani, Kouji H. Harada

**Affiliations:** Department of Health and Environmental Sciences, Kyoto University Graduate School of Medicine, Yoshida, Kyoto, Japan

**Keywords:** Perfluoroalkyl carboxylic acid, Eicosapentaenoic acid-to-arachidonic acid ratio, Fish consumption, Japanese

## Abstract

**Background:**

Per- and polyfluoroalkyl substances (PFASs) are highly fluorinated organic compounds that have been widely used in industry during the past few decades. The main exposure routes for PFASs are thought to be the diet, drinking water, and dust. In this study, we aimed to evaluate the relationship between perfluoroalkyl carboxylic acids (PFCAs, members of the PFAS family) and the plasma eicosapentaenoic acid-to-arachidonic acid ratio (EPA/AA), a biological indicator of seafood intake, to determine whether seafood intake may represent a means of exposure to PFASs in the Japanese population.

**Methods:**

We performed a cross-sectional study using 131 plasma samples collected from residents of Kyoto, Japan in 2013 and held in the Kyoto University biological sample bank. The concentrations of perfluoroheptanoic acid (PFHpA), perfluorooctanoic acid (PFOA), perfluorononanoic acid (PFNA), perfluoroundecanoic acid (PFDA), perfluoroundecanoic acid (PFUnDA), perfluorododecanoic acid (PFDoDA), perfluorotridecanoic acid (PFTrDA), EPA, and AA were quantified by gas chromatography-mass spectrometry, and multiple linear regression was used to analyze the results.

**Results:**

In multiple linear regression analyses with age and eGFR, PFOA showed a significant positive linear association with age (*p* = 0.0005); PFHpA showed a significant negative linear association with estimated glomerular filtration rate (eGFR; *p* = 0.0338); and PFHpA, PFOA, PFNA, PFUnDA, and PFDoDA exhibited significant positive linear associations with EPA/AA (*p* = 0.0358, 0.0056, 0.0242, <0.0001, and <0.0001, respectively). Because only PFHpA and PFOA were associated with smoking, their concentrations were examined again with smoking variable included and neither showed an association with smoking habit. PFOA showed a significant linear association with EPA/AA ratio (*p* = 0.0072), but PFHpA did not (*p* = 0.051).

**Conclusions:**

The plasma concentrations of PFOA, PFNA, PFUnDA and PFDoDA significantly associated with the EPA/AA ratio in residents of Kyoto.

## Graphical Abstract

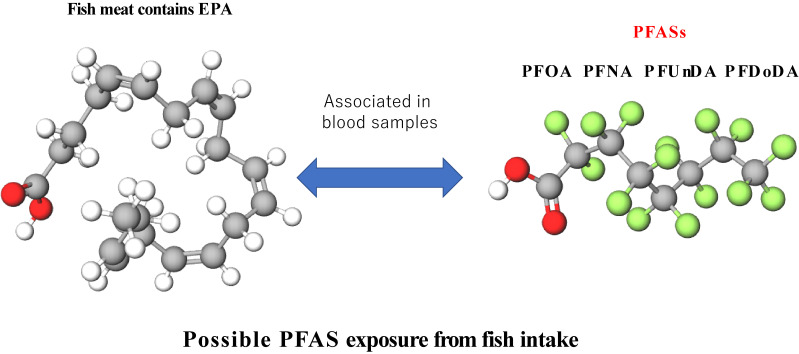

## Background

Per- and polyfluoroalkyl substances (PFASs) are a group of persistent organic chemicals that contain a carbon chain in which hydrogen atoms are replaced by fluorine atoms [1]. The carbon-fluorine bonding is extremely strong, which means that PFASs are highly stable and persistent chemicals. These chemicals have hydrophobic and hydrophilic moieties, predisposing them to form a layer between aqueous and organic solvents or liquids and solid surfaces [1]. In recent decades, PFASs have been widely used in the textile, paper-making, and fire-fighting industries, as well as for other industrial applications and in consumer products [2].

PFASs are ubiquitously detected in the air, water, and soil [3]; therefore, the main exposure routes of the population to PFASs are presumed to be diet [4], drinking water [5], and dust [6]. PFASs such as perfluorooctanoic acid (PFOA) and perfluorooctane sulfonate (PFOS) are very persistent and tend to bioaccumulate [7], as do their homologs with longer chain lengths (LC-PFCAs). As for other organic pollutants [8], seafood consumption might represent a major exposure route. For example, a study conducted in the USA demonstrated that pregnant women that consume a lot of fish and shellfish have high serum PFAS concentrations [9]. In addition, a cohort study of pregnant women conducted in Spain which showed that the consumption of 5.6 servings of seafood a week was associated with higher plasma PFOS, PFOA, and perfluorononanoic acid (PFNA) concentrations than the lowest intake group [10]. Associations have also been reported for selected PFASs, PFOS, and PFOA that can be obtained through the consumption of fish in Japanese residents [11]. Various studies have demonstrated that fish consumption is the primary source of exposure, and this source would be expected to be especially important in Japanese people because of their high seafood consumption.

To evaluate the contribution of seafood consumption to PFAS exposure, conventional biomarkers have previously been measured. Eicosapentaenoic acid (EPA) is a long-chain polyunsaturated omega-3 fatty acid that is most commonly found in fish, and arachidonic acid is an omega-6 fatty acid [12, 13]. The EPA/AA ratio is high in people who consume a lot of fish [14], and this has beneficial effects on the incidences of cardiovascular disease [15], stroke [16], and diabetes [17]. However, the relationship of the EPA/AA ratio with serum PFAS concentration has not been thoroughly investigated in the Japanese population. Therefore, in the present study, we evaluated the relationships between the plasma concentrations of perfluoroalkyl carboxylic acids (PFCAs, members of the PFAS family) and the EPA/AA ratio, which is a biological indicator of seafood intake [18], to determine whether seafood intake may represent an exposure pathway for PFASs in the Japanese population.

## Methods

### Study sample

Uji city is located in the southern area of Kyoto Prefecture, about 15 km south of Kyoto City, Japan. A 1-day event was held as part of health promotion program at a community center in November 2013. Flyers were distributed to each household in the city (73,430 households and 187,577 persons), that described the timing of the event and the measurements that would be made. Five hundred citizens visited this program, and they were informed on this study in the main session that all the visitors attended. Inclusion criteria was adult without blood coagulation disorder, living in Kyoto prefecture. Persons with end-stage renal disease were excluded due to possible difference in toxicokinetics of PFASs. Finally, one hundred thirty-one people were recruited to this study. They provided their written informed consent prior to participation. Demographic data and clinical history were obtained through face-to-face interviews using a structured questionnaire. This study was approved by the Ethics Committee of the Kyoto University Graduate School of Medicine (approval number R1478).

### Sample collection

Blood samples of 10 mL were collected into two 5-mL vacuum collection tubes (Venoject EDTA-K; Terumo, Tokyo, Japan) from a median cubital vein by a physician or nurse. Plasma samples were obtained by centrifugation at 1,000 × *g* for 15 min and stored at −30 °C at the Kyoto Human Specimen Bank until analyzed [19].

### Chemical analysis of PFASs and fatty acids

The concentrations of seven PFCAs were measured: perfluoroheptanoic acid (PFHpA), PFOA, PFNA, perfluorodecanoic acid (PFDA), perfluoroundecanoic acid (PFUnDA), perfluorododecanoic acid (PFDoDA), and perfluorotridecanoic acid (PFTrDA); and those of EPA and AA were also analyzed.

Samples were extracted using a previously reported method [20], involving the addition of 0.5 mL of 0.5 M tetrabutyl ammonium solution (pH 10) and 10 µL of surrogate standard solution (0.1 mg/L MPFAC-MXA, Wellington Laboratories, Guelph, ON, Canada; and 0.1 g/L docosahexaenoic acid-d5, Cayman Chemical, Ann Arbor, MI, USA) to each 0.5 mL plasma sample. Extraction with 1 mL methyl t-butyl ether was conducted twice, and the organic layer obtained was dried. The extracted PFCAs and fatty acids were derivatized for gas chromatography using a previously reported method [21]. Subsequently, 0.1 M pentafluorobenzyl bromide/0.1 M 18-crown-6-ether acetone solution, 1–3 mg of potassium hydrogen carbonate powder, and the internal standard solution 11H-perfluoroundecanoic acid (10 ng) were added, and the mixture was heated at 60 °C for 60 minutes to obtain pentafluorobenzyl ester derivatives. PFCAs and fatty acids esters were separated using an 6890GC and HP-5MS (Agilent Technologies, Santa Clara, CA, USA). Splitless injections (1 µL) were done with an injector temperature of 220 °C, and the split vent was opened after 1.5 min. The initial oven temperature was 70 °C for 2 min, then increased to 100 °C at 20 °C per min, and then to 280 °C at 30 °C per min.

The PFCA and fatty acid concentrations were measured using chemical ionization-mass spectrometry (negative ionization mode, 5973MSD, Agilent Technologies), according to the m/z ratio of [M-C_7_H_2_F_5_]^−^. The reagent gas was methane (99.9999% purity; Air Liquide Japan Ltd.) and supplied at 2 mL per min. The ion source temperature was maintained at 150 °C.

### Assessment of kidney function

Kidney function was evaluated to determine its effects on the plasma PFCA concentrations, because renal clearance is likely to be the primary elimination route for PFASs [22, 23]. An enzymatic assay using creatinine amidohydrolase was used to determine the plasma creatinine concentration. The eGFR (mL/min/1.73 m^2^) was calculated using the age, sex, and serum creatinine concentrations of the participants [24].

### Statistical analysis

Continuous variables were summarized with means, standard deviations and medians. To compare plasma PFCA concentrations, concentrations below the limits of detection (LODs) were set as LOD/ √2 . Calculated values between LODs and limits of quantification were used as they were since the most of values were larger than the limits. Two-tailed *p*-values < 0.05 were considered to represent statistical significance. However, it should not be interpretated as threshold of scientific significance [25]. Differences in the means were examined using ANOVA. Multiple linear regression was used to evaluate the relationships between plasma PFCA levels and other variables. Age and eGFR were considered for potential contributing factors on plasma PFCA levels while other variables were additionally considered if they were associated with plasma PFCA levels in univariate analyses. The analyses were performed using JMP Pro Statistical Software, version 16 (SAS institute, Cary, NC, USA).

## Results

The characteristics of the participants and their concentrations of PFCAs are shown in Tables 1 and 2. The participants comprised 37 men and 94 women, whose mean age was 63 years. Only nine participants were current smokers, but 19 had smoked in the past. Almost half of the participants (n = 64) consumed alcohol. More than 70% had no history of hypertension, dyslipidemia, liver disease, kidney disease, cardiac disease, or diabetes. Their mean serum creatinine concentration, eGFR, and EPA/AA ratio were 0.68 mg/dL, 77.5 mL/min/1.73 m^2^, and 0.59, respectively.

**Table 1:** Characteristics of participants from whom plasma samples were collected in 2013

**Characteristic**		**n (%) or Mean (SD), median**
Total number		131 (100%)^a^
Age (years)		63 (15), 67
Sex	Male	37 (28%)
	Female	94 (72%)
Height (cm)		157.7 (8.5), 156.3
Body mass (kg)		53.5 (9.7), 51.3
Smoking	No	102 (78%)
	Past history	19 (15%)
	Yes	9 (7%)
Alcohol consumption	No	52 (40%)
	Past history	14 (11%)
	Yes	64 (49%)
Serum creatinine (mg/dL)		0.68 (0.15), 0.64
eGFR (mL/min/1.73 m^2^)		77.5 (16.7), 73.7
EPA/AA ratio		0.59 (0.31), 0.56
Disease histories		
Hypertension	Yes	38 (30%)
	No	92 (70%)
Dyslipidemia	Yes	25 (20%)
	No	105 (80%)
Diabetes	Yes	2 (2%)
	No	128 (98%)
Liver disease	Yes	2 (2%)
	Past history	4 (3%)
	No	124 (95%)
Kidney disease	Yes	4 (3%)
	Past history	2 (2%)
	No	124 (95%)
Cardiac disease	Yes	12 (9%)
	Past history	1 (1%)
	No	117 (89%)

SD, standard deviation; EPA/AA, eicosapentaenoic acid-to-arachidonic acid ratio; eGFR, estimated glomerular filtration rate. ^a^Only the age and sex of one of the participants were available. One participant did not provide the questionnaire disease histories, smoking and drinking.

**Table 2:** Plasma concentration of perfluoroalkyl carboxylic acids (pg/mL)

	**PFHpA**	**PFOA**	**PFNA**	**PFDA**	**PFUnDA**	**PFDoDA**	**PFTrDA**
Percentage of detection frequency	99.2%	100%	100%	100%	100%	98.5%	100%

LOD	15	80	20	20	20	30	50

Q_5_	30	1170	934	228	338	48	112
Q_25_	43	2940	1880	460	610	85	148
Q_50_	57	4080	2510	659	895	124	183
Q_75_	77	6340	3380	942	1120	168	218
Q_95_	124	9030	6790	1820	2220	314	304
Mean	63.4	4630	3020	869	998	141	191
SD	30.0	2450	2200	1130	602	78.9	72.0

LOD, limit of detection; PFHpA, perfluoroheptanoic acid; PFOA, perfluorooctanoic acid; PFNA, perfluorononanoic acid; PFDA, perfluoroundecanoic acid; PFUnDA, perfluoroundecanoic acid; PFDoDA, perfluorododecanoic acid; PFTrDA, perfluorotridecanoic acid; Q, percentile; SD, standard deviation. Data of 131 participants was summarized.

All the PFCAs, except PFHpA and PFDoDA, were detected in all of the participants. However, PFHpA was detected in 99.2% and PFDoDA was detected in 98.5% of the participants, implying that almost seven PFCAs were detected in all the participants. PFOA was present at the highest concentrations, followed by PFNA. Table 2 shows the distributions of the serum PFCAs concentrations. Means of them were comparable to medians.

Next, the relationships between the plasma PFCA concentrations and demographic factors were evaluated. Table 3 shows the Pearson’s correlation coefficients and contingency tables for the relationships of PFCA concentrations with age, sex, smoking, alcohol consumption, eGFR, and EPA/AA ratio. All of the PFCAs showed positive correlations with age, except for PFTrDA. PFHpA and PFOA showed significant associations with smoking. However, none showed associations with sex or alcohol consumption. Most of the PFCAs showed significant negative linear associations with eGFR, but PFDA and PFTrDA did not. All of the detected PFCAs showed significant positive linear associations with the plasma EPA/AA ratio.

**Table 3:** Relationships of plasma perfluoroalkyl carboxylic acid concentrations with age, sex, smoking, drinking, eGFR, and EPA/AA

	**PFHpA**	**PFOA**	**PFNA**	**PFDA**	**PFUnDA**	**PFDoDA**	**PFTrDA**
	Pearson’s correlation coefficients						

**Age**							
***r***	0.293	0.531	0.300	0.204	0.293	0.329	0.137
***p***	0.0007	<0.0001	0.0005	0.0196	0.0007	0.0001	0.12
**eGFR**							
***r***	−0.323	−0.414	−0.193	−0.103	−0.213	−0.224	0.021
***p***	0.0002	<0.0001	0.0281	0.24	0.0152	0.0104	0.81
**EPA/AA**							
***r***	0.260	0.378	0.286	0.209	0.432	0.442	0.205
***p***	0.0027	<0.0001	0.0009	0.0168	<0.0001	<0.0001	0.0186


	Mean (SD)						

**Sex**							
**Male**	60.8 (37.0)	4100 (2630)	3020 (3020)	1020 (1970)	920 (568)	131 (66.6)	203 (101)
**Female**	64.5 (26.9)	4830 (2360)	3020 (1800)	808 (529)	1030 (615)	144 (83.3)	187 (56.8)
***p***	0.52	0.12	0.99	0.33	0.35	0.41	0.25
**Smoking**							
**Never**	67.1 (32.0)	4940 (2500)	3140 (2340)	924 (1220)	1050 (633)	147 (82.8)	195 (76.3)
**Past history**	52.6 (14.4)	3620 (2000)	2650 (1630)	729 (863)	853 (434)	130 (57.2)	178 (57.1)
**Current**	46.9 (21.0)	3430 (2010)	2660 (1690)	603 (317)	796 (500)	95.4 (55.9)	183 (43.4)
***p***	0.0336	0.0294	0.59	0.60	0.25	0.13	0.61
**Alcohol consumption**							
**Never**	67.8 (30.8)	4850 (2500)	3220 (1940)	897 (727)	1110 (699)	155 (91.6)	183 (55.1)
**Past history**	64.9 (32.1)	4630 (2380)	2460 (1210)	606 (313)	767 (297)	117 (55.7)	187 (32.4)
**Current**	59.9 (29.0)	4480 (2460)	3010 (2550)	913 (1470)	967 (554)	136 (70.5)	200 (88.2)
***p***	0.37	0.72	0.52	0.65	0.14	0.21	0.44

PFHpA, perfluoroheptanoic acid; PFOA, perfluorooctanoic acid; PFNA, perfluorononanoic acid; PFDA, perfluoroundecanoic acid; PFUnDA, perfluoroundecanoic acid; PFDoDA, perfluorododecanoic acid; PFTrDA, perfluorotridecanoic acid; SD, standard deviation; EPA/AA, eicosapentaenoic acid-to-arachidonic acid ratio; eGFR, estimated glomerular filtration rate. Data are presented as mean and SD for categorical variables. Data of 131 participants was analyzed. One participant was excluded for smoking and drinking. Pearson’s correlation coefficients, *r* were used to evaluate relationships between continuous variables and tested by *t*-distribution. Differences in the means between groups were examined using ANOVA.

To adjust for the potentially confounding effects of age and eGFR, multiple linear regression analysis was performed. This showed that the plasma PFOA concentration significantly correlated with age; that PFHpA significantly correlated with eGFR; and that PFHpA, PFOA, PFNA, PFUnDA, and PFDoDA significantly positively correlated with the EPA/AA ratio (Table 4).

**Table 4:** Associations between plasma PFCA concentrations and EPA/AA adjusted with age and eGFR

	**β, point estimate (95% confidence interval)**						

	**PFHpA**	**PFOA**	**PFNA**	**PFDA**	**PFUnDA**	**PFDoDA**	**PFTrDA**
Age	0.08 (−0.42, 0.58)	64.9 (28.8, 101)	34.9 (−2.05, 71.8)	15.1 (−4.58, 34.7)	5.05 (−4.55, 14.7)	0.93 (−0.32, 2.17)	1.04 (−0.21, 2.28)
*p*	0.75	0.0005	0.06	0.13	0.3	0.14	0.1

eGFR	−0.50 (−0.96, −0.04)	−14 (−47.3, 19.3)	2.6 (−31.5, 36.7)	5.15 (−13.0, 23.3)	−1.73 (−10.6, 7.14)	−0.11 (−1.26, 1.04)	0.98 (−0.17, 2.13)
*p*	0.0338	0.41	0.88	0.58	0.7	0.85	0.09

EPA/AA	18.4 (1.24, 35.6)	1770 (530, 3020)	1470 (195, 2750)	564 (115, 1240)	743 (411, 1070)	95.7 (52.7, 139)	39.1 (−3.93, 82.1)
*p*	0.0358	0.0056	0.0242	0.1	<0.0001	<0.0001	0.07

Data of 131 participants was analyzed.

Regression coefficients (*β*) are expressed as point estimate (95% confidence interval) in multiple linear regression analysis. PFCA, perfluoroalkyl carboxylic acid; PFHpA, perfluoroheptanoic acid; PFOA, perfluorooctanoic acid; PFNA, perfluorononanoic acid; PFDA, perfluoroundecanoic acid; PFUnDA, perfluoroundecanoic acid; PFDoDA, perfluorododecanoic acid; PFTrDA, perfluorotridecanoic acid; EPA/AA, eicosapentaenoic acid-to-arachidonic acid ratio; eGFR, estimated glomerular filtration rate.

Because only PFHpA and PFOA were associated with smoking (Table 3), we further evaluated the relationships of PFHpA and PFOA with age, smoking, eGFR, and EPA/AA ratio (Table 5). This analysis showed that PFHpA was not significantly associated with age or smoking, and that PFOA no longer significantly correlated with eGFR. The plasma PFOA concentration correlated with EPA/AA ratio and the plasma PFHpA concentration showed a weak correlation with EPA/AA ratio.

**Table 5:** Relationships of the plasma PFHpA and PFOA concentrations with EPA/AA, age, smoking, and eGFR

	**β, point estimate (95% confidence interval)**	

	**PFHpA**	**PFOA**
Age	0.06 (−0.44, 0.55)	63.29 (27.3, 99.3)
*p*	0.83	0.0007
Smoking (reference: Never)		
Current smoker	−3.56 (−17.0, 9.85)	52.0 (−921, 1020)
*p*	0.60	0.92
Ex-smoker	−3.63 (−14.3, 7.04)	−437 (−1210, 336)
*p*	0.50	0.27
eGFR	−0.46 (−0.92, −0.00)	−12.45 (−45.9, 21.0)
*p*	0.0481	0.46
EPA/AA	17.2 (−0.05, 34.4)	1,730 (476, 2980)
*p*	0.051	0.0072

Data of 130 participants was analyzed.

Regression coefficients (*β*) are expressed as point estimate (95% confidence interval); PFHpA, perfluoroheptanoic acid; PFOA, perfluorooctanoic acid; EPA/AA, eicosapentaenoic acid-to-arachidonic acid ratio; eGFR, estimated glomerular filtration rate.

## Discussion

In the present study, we found that several factors were associated with plasma PFCA concentrations in Japanese people in Kyoto. Notably, the EPA/AA ratio showed significant correlations with five of the seven PFCAs assessed in multivariable regression analyses (PFHpA, PFOA, PFNA, PFUnDA, and PFDoDA).

The plasma concentrations of PFCAs, and particularly of PFOAs, were similar to those identified in a previous study conducted in Japan [26]. Of the LC-PFCAs, those with odd-numbered carbon chains (PFNA, PFUnDA, and PFTrDA) were present at higher concentrations than those with even-numbered carbon chains (PFDA, and PFDoDA). The concentration of PFCAs measured in the present study were also comparable to those measured in studies conducted in Belgium [27] and the USA [28]. In all of these studies, PFOAs were found to be the principal representatives of the PFCAs.

Age was found to be significantly positively correlated with the concentrations of PFCAs, except for that of PFTrDA (Tables 3–5). This might be because of the long biological half-life of PFCAs. According to Wang et al., the estimated industrial emission of PFOA during recent years was much higher than that of other PFCAs [7]. However, there are few data regarding the annual production and emissions of each PFCA in Japan; therefore, further research is required to determine individual exposures to these PFCAs.

PFCAs, except PFDA and PFTrDA, were found to negatively correlate with eGFR. This may suggest that PFCA is excreted in the urine. However, further analysis showed that only the concentration of PFHpA negatively correlated with kidney function. Shorter-chain PFCAs are more likely to be eliminated through renal clearance [23, 29]. Indeed, a longitudinal study by Lin et al. that evaluated the relationship of PFAS with eGFR in patients with diabetes showed that after 14 years of follow-up, plasma PFAS concentrations were inversely correlated with eGFR [30].

Previously, Yamaguchi et al. demonstrated associations of plasma PFOS and PFOA concentrations with the EPA/AA ratio and seafood intake in Japan [11]. This study showed that the consumption of broth-boiled fish, sliced raw fish, and coastal fish was significantly positively correlated with circulating PFOS concentrations. In addition, in a previous study, the concentrations of PFCAs in Pacific cod samples were measured in several parts of Japan’s coastal and Korean waters [31]. The total concentrations of PFCAs were found to range from 819 to 1,710 pg/g wet weight of cod in Japan and 288 to 892 pg/g wet weight in Korea. Because seafood may represent an important dietary source of PFCAs, high concentrations of PFCAs in cod from Japanese and Korean waters may affect human dietary exposure and the circulating concentrations of PFCAs [31]. Although fish consumption might be one of the routes of PFAS exposure, fish intake is beneficial for our health [32]. A recent meta-analysis showed a possible link between fish intake and acute myeloid leukemia, but the association was not observed in Japanese [33]. Thus, the benefits of fish intake should not be undermined by this study results.

There were several limitations to the present study. First, the participants all lived in Kyoto, and therefore the described relationships cannot necessarily be generalized to other regions. The recruitment was based on the health promotion program and there should be a selection bias in the participants. Second, we did not take other possible routes of exposure into account, such as drinking water [34], personal care products [35], and indoor dust [36], which might have confounded the relationships with EPA/AA ratio. We evaluated the influence of other covariates, such as sex, smoking, and alcohol consumption on plasma PFCA concentrations (Table 3). A previous study showed that the link between PFASs and thyroid function is modified by smoking. However, there are few data regarding the relationship between smoking and the concentrations of PFASs [37]; thus, the potential effects of such habits should be further evaluated. Third, the age distribution of the participants was skewed toward older individuals, and we did not collect samples from children or young people. This is important because fish consumption is lower among the younger generation [38], and therefore the identified relationships might be weaker in younger people. Fourth, we did not have access to previous exposure data in this cross-sectional study. The small sample size and cross-sectional study design in this study were challenging to reproducibility. Further prospective studies with a large sample size are warranted.

## Conclusions

Several plasma PFCA concentrations, and specifically those of PFOA, were found to positively associate with the EPA/AA ratio. In addition, PFHpA was found to negatively associate with eGFR. Seafood intake may represent an exposure pathway for these PFASs in the study population. However, we were unable to assess all the potential covariates that might affect the identified relationships with PFAS concentrations, and the small sample size with cross-sectional study design require confirmation in future studies. It is noted that the benefits of fish intake should not be undermined by this study results.

## Abbreviations

PFASs: per- and polyfluoroalkyl substances
PFCAs: perfluoroalkyl carboxylic acids
PFHpA: perfluoroheptanoic acid
PFOA: perfluorooctanoic acid
PFNA: perfluorononanoic acid
PFDA: perfluorodecanoic acid
PFUnDA: perfluoroundecanoic acid
PFDoDA: perfluorododecanoic acid
PFTrDA: perfluorotridecanoic acid
EPA/AA: eicosapentaenoic acid-to-arachidonic acid ratio
eGFR: estimated glomerular filtration rate
LODs: limits of detection

## Declarations

### Availability of data and material

The data that support the findings of this study are available from the corresponding author upon reasonable request.
